# Bovine Coronavirus Infects the Respiratory Tract of Cattle Challenged Intranasally

**DOI:** 10.3389/fvets.2022.878240

**Published:** 2022-04-29

**Authors:** Katelyn R. Soules, Michael C. Rahe, Lisa Purtle, Craig Moeckly, Paul Stark, Clay Samson, Jeffrey P. Knittel

**Affiliations:** ^1^Merck Animal Health, De Soto, KS, United States; ^2^Department of Veterinary Diagnostic and Production Animal Medicine, Iowa State University, Ames, IA, United States

**Keywords:** bovine coronavirus, respiratory infection, challenge model, histopathology, bovine respiratory disease

## Abstract

Bovine Coronavirus (BCoV) is a member of a family of viruses associated with both enteric and respiratory diseases in a wide range of hosts. BCoV has been well-established as a causative agent of diarrhea in cattle, however, its role as a respiratory pathogen is controversial. In this study, fifteen calves were challenged intranasally with virulent BCoV in order to observe the clinical manifestation of the BCoV infection for up to 8 days after initial challenge, looking specifically for indication of symptoms, pathology, and presence of viral infection in the respiratory tract, as compared to six unchallenged control calves. Throughout the study, clinical signs of disease were recorded and nasal swabs were collected daily. Additionally, bronchoalveolar lavage (BAL) was performed at 4 days Post-challenge, and blood and tissue samples were collected from calves at 4, 6, or 8 days Post-challenge to be tested for the presence of BCoV and disease pathology. The data collected support that this BCoV challenge resulted in respiratory infections as evidenced by the isolation of BCoV in BAL fluids and positive qPCR, immunohistochemistry (IHC), and histopathologic lesions in the upper and lower respiratory tissues. This study can thus be added to a growing body of data supporting that BCoV is a respiratory pathogen and contributor to respiratory disease in cattle.

## Introduction

Coronaviruses (CoVs) are a large group of positive-sense RNA, enveloped viruses that cause a variety of diseases in numerous mammalian and avian hosts, including humans, cattle, pigs, chickens, cats, mice, and many other species (1, 2). Depending on the host and strain of coronavirus, the site of infection and disease manifestation can vary. In pigs, coronavirus infections mostly lead to enteric symptoms, but also have the ability to infect the nervous system (1, 3). A virulent feline coronavirus can cause systemic disease in cats by infecting macrophages and lymphatic tissue (3, 4). In chickens, some strains of coronavirus can affect the urogenital tract causing renal disease and diminished egg production (3). In 2008, Mihindukulasuriy et al. isolated coronavirus from the liver of a whale found deceased with respiratory disease and acute liver failure (5). As demonstrated by the recent pandemic strain of coronavirus, SARS-CoV 2, also known as COVID-19, these viruses have the potential to spread rapidly and cause a high degree of pathogenicity with the potential to be lethal (6–8).

Bovine coronavirus (BCoV) is a well-known cause of enteric disease in cattle, notably causing illnesses such as “winter dysentery” (1, 9–16). These illnesses can cause weight loss, dehydration, decreased milk production, depression, and potentially death, all of which can lead to significant economic loss (3). In addition to cattle, BCoV can spread to a variety of other ruminants, including elk, deer, and camels (17, 18). While BCoV is widely accepted as an enteric pathogen with a significant impact on the cattle industry, it is not often clinically regarded as a respiratory pathogen (19, 20). Furthermore, there is currently a shortage of BCoV challenge studies which effectively reproduce clinical respiratory disease and show gross and microscopic pathology (21–23).

In this study, fifteen calves were administered virulent BCoV intranasally (IN). The six control calves were not challenged but were housed in a separate room in the same building as the challenged calves. The data collected support that this BCoV challenge in calves resulted in clinical disease and respiratory infections as evidenced by the isolation of BCoV in BAL fluids and nasal swabs, as well as positive qPCR, IHC, and histopathology in the lungs and other tissues of the respiratory tract.

## Materials and Methods

**Animals**. Twenty-one newborn colostrum deprived (CD), single-sourced Holstein calves born within 48 hours of each other were received in a single shipment. All calves were ~4 weeks old and of similar weight at the start of the study. Blood samples collected at the time of arrival to the challenge facility to screen for the BoCV and BVDV infections confirmed that all animals were negative for these viral infections at the commencement of the study. Both male and female calves were included in the study. All animals arrived at the challenge facility and were acclimated for 6 days prior to challenge. Calves were bottle-fed a minimum of two quarts of milk replacer twice a day. Once they could be fed with a bucket, they additionally received 16% calf grower, as needed. Water was available *ad libitum*. Calves were randomly assigned to challenge or control groups. Fifteen calves were included in the challenge group (Group 1) and six calves were included in the negative control group (Group 2). All animals were housed in the same building; however, negative control calves were housed in a separate room apart from the challenge calves with solid walls in between. The air in the building goes through MERV30/30 filters prior to entry into the animal housing rooms, with an airflow sink pulling in one direction out of the rooms. Fresh personal protective equipment (PPE) (Tyvek suits, boots, gloves, etc.) were donned in between each animal room with the unchallenged, control calves (Group 2) always being attended to first for feeding, care, clinical observations, sample collection, and all other daily activities. Animal care personell wore disposable masks throughout the study. Challenge calves (Group 1) were randomly split into three subgroups of five calves, and on day 4, 6, or 8 Post-challenge one subgroup of calves was euthanized and the following fresh and fixed tissues were collected at the time of euthanasia: eyelids, nasal turbinates, tonsils, trachea, primary bronchus, distal bronchus, lung, and tracheobronchial lymph node. A cutoff was set for animals with an initial BCoV antibody titer >64 prior to challenge to be excluded from the study. One animal in group 1A was determined to have an antibody titer >64, and thus was substituted with calf #685 on day −1.

**Veterinary care and observation**. The health of the animals was managed by the on-site veterinarians. All animals were examined prior to the start of the study to determine overall health status. No adverse events, severe illness, or distress was observed that required medical intervention throughout the duration of the study. Daily observations for overall health of the animals were performed by the animal care staff prior to initiation of the study and by the study investigator through the duration of the study. No disease symptoms unrelated to BCoV infection were observed throughout the study. All calves were monitored for symptoms of clinical disease, respiratory rate, and rectal temperatures for 3 days prior to challenge and for up to 8 days Post-challenge.

**Challenge material**. The virulent BCoV respiratory challenge strain was MN-1988, originally isolated from a 1-week old calf submitted to the Veterinary Diagnostic Lab in St. Paul, Minnesota. The isolate was propagated in CD calves, reisolated, and then propagated in HRT-18 cells. The challenge virus target dose was 7.0 log_10_ TCID_50_ per calf (6.4 log_10_ TCID_50_ per mL) in a 4 mL dose. Five, 5 mL aliquots of frozen virulent BCoV respiratory challenge strain MN-1988 were quickly thawed and pooled. Twenty milliliters of pooled virus was diluted in Dulbecco's Modified Eagle's Medium (DMEM) (Hyclone) and divided into 4 mL doses within an hour prior to administration to the calves while being kept on ice for the entirety of the time between dilution and administration. The challenge virus was tested for potency immediately after dilution and determined to have a titer of 7.1 log_10_ TCID_50_ per 4 mL dose (6.5 log_10_ TCID_50_ per mL). Administration of the challenge material to the calves was performed intranasally via a Flexineb (BreathEazy, Worchesterchire, UK) equine nebulizer (foal/pony size) until all of the viral-containing fluid had been nebulized, ~2–3 min.

**Blood sample collection and serum neutralization titers**. Blood samples were also collected on the day of euthanasia (day 4, 6, or 8 Post-challenge). Blood was collected in a serum separator tube (10 to 15 mL). The blood samples were incubated at 37 ± 2°C for 30 to 120 min and then centrifuged at 2,000 rpm for 20 min to separate the sera. The sera were heat-treated for 30 min in a 56°C water bath. Serum samples were tested for SN antibody titers to BCoV.

Serum samples were prepared in serial 2-fold dilutions up to 1:8,192 and were tested in triplicate wells. Approximately 50–500 TCID_50_ per well of the BCoV SN virus was added to the diluted serum samples. The plates containing a mixture of the serum samples and virus were incubated for 60 ± 10 min in a 37 ± 2°C in a 4–6% CO_2_ humidified chamber to allow any antibodies present in the serum to neutralize the SN virus. The sera/virus mixtures were then transferred to 96-well tissue culture plates containing confluent monolayers of HRT-18 cells. The test plates were incubated at 37 ± 2°C in a 4–6% CO_2_ humidified chamber for 5 days. The cells were fixed with methanol, reacted with specific anti-BCoV monoclonal antibody at a 1:20,000 dilution in PBS, and stained with an anti-mouse IgG-FITC conjugate (SeraCare; Milford, MA). Cells were examined with a fluorescent microscope and scored for the presence or absence of fluorescence. Titers were calculated using the Spearman-Karber 50% end point method and expressed as log_10_ TCID_50_ per mL.

**Nasal swab collection and processing**. Two sterile polyester swabs (one per nostril) were used to collect nasal secretions from each animal on day −1 and day 1 Post-challenge through the day of euthanasia. The nasal swabs were inserted ~6 inches into each nostril and gently rubbed against the nasal mucosal surface, then both swabs were placed into a tube containing 2.5 mL of cold nasal swab media (DMEM supplemented with gentamicin at 200 ug/mL, penicillin-streptomycin at 100 IU/mL and 100 ug/mL, and amphotericin B at 5.0 ug/mL) and placed on ice. The nasal swabs were then vortexed in the transport media and the fluid was squeezed from the swab by pressing the swab against the wall of the tube. The fluid was centrifuged at 1,000 g for 10 min at 2–8°C. Samples were stored at ≥−70°C before being tested for viral titers.

**Bronchoalveolar lavage**. Fifty milliliters of chilled, sterile PBS was administered to each calf using a 1.8 mm endoscopic microbiology aspiration catheter (MILA International, INC) and endoscope (Whittemore VET-8015). Calves were restrained with their heads extended so that the upper respiratory tract formed a straight line and the catheter was placed into the trachea via the ventral meatus of the nasal passage. Once in the trachea the tube was gently advanced until it reached the bifurcation of the trachea to reach the bronchi. The 50 mL PBS was carefully administered and the liquid immediately re-aspirated using the same tube. The recovered fluid was transferred into a sterile container and held on ice before being centrifuged at 1,000 g for 10 min at 2-8°C. Samples were stored at ≥−70°C before being tested for viral titers.

**Viral titers from collected samples**. 10-fold serial dilutions of the nasal swab or bronchoalveolar lavage (BAL) samples from each animal were added to four wells of a 96-well microtiter plate containing confluent monolayers of HRT-18 cells. The plates were incubated for 5 days at 37 ± 2°C in a humidified 4–6% CO_2_ incubator. The cells were then fixed with methanol, incubated with anti-BCoV monoclonal antibody, and stained with an anti-mouse IgG-FITC conjugate. Cells were examined with a fluorescent microscope and scored for the presence or absences of fluorescence. Virus titers were calculated using the Spearman-Karber 50% end point method and expressed as log_10_ TCID_50_/mL.

**BCoV PCR of tissue samples**. Fresh tissue samples were collected at the time of euthanasia, placed in separate plastic bags, and shipped on ice to the Iowa State University Veterinary Diagnostic Laboratory (ISU VDL). Fresh tissues were processed and analyzed at the ISU VDL as previously described (24). RT-rt PCR primers used were specific for the S gene of BCoV.

**Histopathology and BCoV IHC**. Tissue samples (nasal turbinates, trachea, bronchus, deeper bronchus, lung, tracheobronchial lymph node, tonsil, and eyelid) collected at the time of euthanasia were immediately placed in a 10% neutral buffered formalin solution and shipped to ISU VDL for H&E and IHC evaluation. The nasal turbinates, trachea, proximal bronchus, and distal bronchus were evaluated and scored for histopathology and IHC by a blinded diagnostic pathologist (Rahe) using an Olympus BX41 microscope. For histopathology scoring of airways, the following grading scheme modified from Larios Mora et al. was used (25). 0 = Normal epithelium with no evidence of inflammatory infiltrates. 1 = Minimal detectable epithelial cell necrosis, loss, or hyperplasia with sparse inflammatory cell (neutrophils, lymphocytes, or plasma cells) infiltrates in the lamina propria. 2 = ~10% of epithelium is necrotic, lost, or hyperplastic with mild inflammatory cell infiltrates. 3 = Between 10 and 50% of epithelium is necrotic, lost, or hyperplastic with moderate inflammatory cell infiltrates. 4 = Between 50 and 100% of the respiratory epithelium is necrotic, lost, or hyperplastic with moderate to severe inflammatory cell infiltrates. The lung, eyelid, tracheobronchial lymph node, and tonsil and were also evaluated for histologic lesions and IHC staining.

IHC for detection of BCoV-specific antigen (nucleocapsid) was performed on fixed tissue as previously described utilizing equal parts of ascites IDs BC26C8.2C, BC22H5.3C, BC22F8.3C at a 1:400 dilution (Reference to be added, accepted manuscript). The following grading scheme was used for IHC scoring: 0 = no signal, 1 = mild signal (rare epithelial cells or leukocytes have intracytoplasmic staining), 2 = moderate signal (multifocal groups of epithelial cells or leukocytes have intracytoplasmic staining) 3 = abundant signal (multifocal to coalescing groups of epithelium or leukocytes have intracytoplasmic staining).

## Results

**Clinical disease observations suggest both respiratory and enteric symptoms**. Calves challenged with BCoV displayed clinical symptoms of disease, most frequently nasal discharge, mild cough, and diarrhea (Supplementary Table S1). The challenge calves (Group 1) started displaying nasal discharge at day 4 Post-challenge, at which time 3 out of 15 (20%) of the calves had moderate discharge. Seven of the 10 (70%) challenge calves that remained in the study until day 6 or 8 Post-challenge had moderate nasal discharge on at least 1 day Post-challenge. Fever (rectal temperatures of 103.0°F or greater) was detected in 5 of 15 animals during the first 4 days of infection. Elevated respiratory rates (>60/min) were observed in 3 of 15 calves over that same time period (Supplementary Tables S2, S3). Only one calf (ID #647) displayed an elevated respiratory rate as well as a fever; however, the elevated respiratory rate preceded the fever by 1 day. Clinical symptoms peaked at day 5 Post-challenge with 9 out of 10 (90%) remaining challenge calves displaying some symptoms of BCoV infection, most commonly nasal discharge (Supplementary Table S1). Notably, on day 6 Post-challenge 60% of the remaining challenge calves were observed to have a mild cough, while none of the control calves had coughing symptoms throughout the duration of the study. Diarrhea was observed in a control calf on day 3 and another on day 6, subsequently leading to the development of minor clinical signs.

**Viral shedding detected in nasal secretions and bronchoalveolar lavage fluid show presence of BCoV in the respiratory tract**. BCoV was isolated from nasal swab secretions of all challenge calves starting from day 1 Post-challenge through day 7 Post-challenge (Table 1 and Supplementary Table S4). On the final day of the study, day 8 Post-challenge, 2 of the remaining 5 challenge calves (40%) still had detectable BCoV titers in nasal secretions. BCoV infection titers peaked at day 5 Post-challenge for the challenge group with an average titer of 6.6 log_10_ TCID_50_/mL in the nasal secretions. Control calves maintained negative results for BCoV isolation until day 6 Post-challenge. By day 8 Post-challenge, both of the remaining control calves had positive nasal swab titers for BCoV.

**Table 1:** Average nasal swab virus shedding titers (Log_10_ TCID_50_/mL).

**Group**	**Pre-Challenge (Day −1)**	**Day 1**	**Day 2**	**Day3**	**Day 4**	**Day 5**	**Day 6**	**Day 7**	**Day 8**
Challenge	0.0	3.7	6.2	6.4	6.0	6.6	5.5	4.0	1.4
Control	0.0	0.0	0.0	0.0	0.0	0.0	0.8	4.6	5.8

Bronchoalveolar lavage (BAL) fluid was collected from all calves on day 4 Post-challenge. Briefly, using an endoscope, a catheter was gently advanced until it reached the bifurcation of the trachea and sterile PBS administered and immediately recollected. BCoV was isolated from BAL fluid from all but one challenge calf (Group 1) (93.3%) with an average viral titer of 3.52 log_10_ TCID_50_/mL. Only one out of six control calves (Group 2) (16.7%) had a BCoV positive BAL sample (BCoV titer of 2.5 log_10_ TCID_50_/mL). Isolation of BCoV from the BAL fluid and the nasal swab samples are evidence for viral infection of the respiratory tract for at least 7 days Post-challenge in the calves.

Supplementary Figure S1 shows a representative image taken using the endoscope at the time of the BAL of the terminal bronchi of a challenge calf, 4 days Post-challenge. This image shows a mass of mucous and tissue debris on the luminal wall of the bronchi, which was a typical observation in the respiratory tracts of the challenge animals during this procedure along with tissue debris that was collected with the lavage fluid. None of the control calves had mucous build up or debris apparent at the time of BAL.

**Serum neutralization antibody titers suggest an immune response developing toward the resolution of BCoV infection**. All calves included in the study had antibody titers ≤ 1:64 to BCoV prior to challenge, meeting the predetermined cutoff for being seronegative. Control calves maintained seronegative BCoV antibody titers throughout the study. On day 8 Post-challenge, 3 out of 5 challenge calves had antibody titers of 128, resulting in an overall geometric mean SN titer (GMT) of 90.5 indicative of the initiation of a humoral immune response specific to BCoV (Table 2 and Supplementary Table S5).

**Table 2:** Summary of serum neutralization antibody titers (Geometric mean SN titer^*^).

**Group**	**Pre-challenge**	**Day 4**	**Day 6**	**Day 8**
Challenge	41.0	42.2	48.5	90.5
Control	57.0	45.3	32.0	45.3

* *For determination of the GMT, any sample with an antibody titer <64 was assigned a value of and was recorded as 32*.

**Figure 1:**
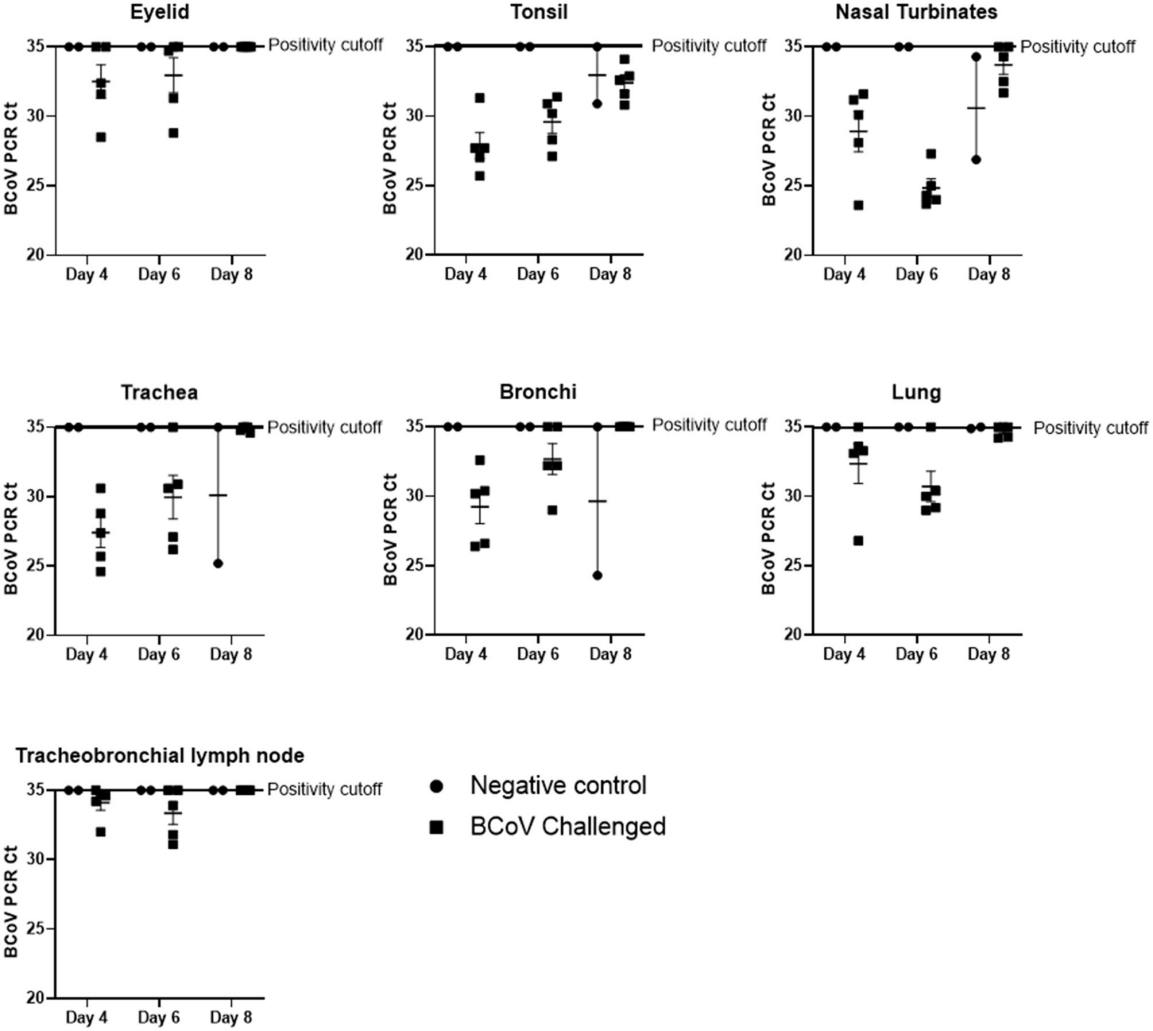
BCoV RT-rtPCR results at days 4, 6, or 8 Post-challenge. All tissue types submitted (nasal turbinate, eyelid, tonsil, trachea, lymph node, bronchioles, and lung) tested positive (CT counts <35) for BCV by qPCR in some or all of the challenge calves (100% of the tonsil samples, 87% of the nasal turbinate, 73% of the trachea, 67% of the lung, 53% of the bronchiole, 47% of the lymph node, and 40% of the eyelid samples). Only two of the six control calves (the calves euthanized on day 8 of the study) had qPCR positive tissues at the time of euthanasia.

**BCoV nucleic acids detected by RT-rtPCR in respiratory tract tissues**. Fresh tissue samples were taken at the time of euthanasia and submitted to a diagnostic lab for RT-rtPCR to evaluate the presence of BCoV. The mean BCoV nucleic acid levels of challenged (Group 1) animals were particularly high in the nasal turbinates, tonsil, lung, and trachea on days 4 and 6 Post-challenge (Figure 1). However, by day 8 Post-challenge only the tonsil and nasal turbinates still had detectable BCoV nucleic acid. Six out of ten challenged calves (Group 1) were PCR positive in the eyelid at days 4 and 6 Post-challenge, though all day 8 Post-challenge animals were negative. Similarly, the tracheobronchial lymph node had positive Ct counts in seven of ten calves at days 4 and 6 Post-challenge, with all animals negative by day 8. On day 8 Post-challenge, at least one negative control (Group 2) calf had a positive Ct count in all evaluated tissues besides the tracheobronchial lymph node and eyelid.

**Figure 2:**
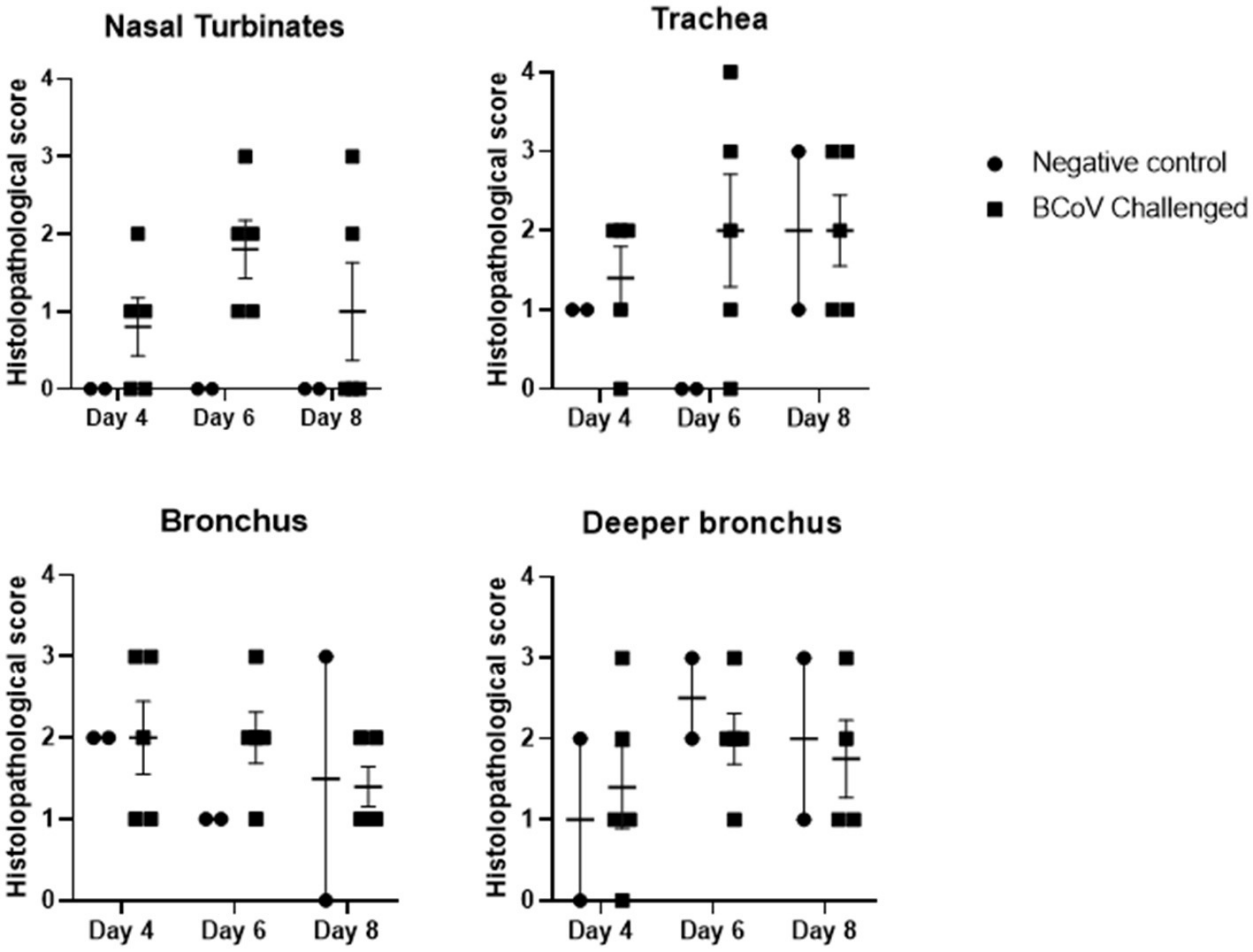
Histopathology scores of fixed tissues at days 4, 6, or 8 Post-challenge. Tissues evaluated from challenge calves showed increased pathology in the nasal turbinates and trachea when compared to the control calves. Differences in tissue pathology were less apparent in bronchus samples between the two groups. 1 = Minimal detectable epithelial cell necrosis, hyperplasia, and sparse inflammatory cell (neutrophils, lymphocytes, or plasma cells) infiltrates in the lamina propria. 2 = Approximately 10% of epithelium is necrotic or hyperplastic with mild inflammatory cell infiltrates. 3 = Between 10 and 50% of epithelium is necrotic or hyperplastic with moderate inflammatory cell infiltrates. 4 = Between 50 and 100% of the respiratory epithelium is necrotic or hyperplastic with moderate to severe inflammatory cell infiltrates.

**Histopathology evaluation and BCoV direct detection by IHC in respiratory tract tissues**. At the time of euthanasia, upper and lower respiratory tract tissue samples were stored in formalin and submitted to a diagnostic laboratory for immunohistochemistry (IHC) and histopathology evaluation. The evaluated airways frequently displayed some degree of epithelial attenuation and inflammatory infiltrate with most severe lesions in the tracheas of challenged calves at days 6 and 8 Post-challenge (Figures 2, **4C**). The nasal turbinates of all challenged calves showed some level of pathology at day 6 Post-challenge, displaying epithelial erosion or ulceration with infiltration of the submucosa by lymphocytes, plasma cells and neutrophils (**Figure 4D**). Epithelium of bronchi from both challenged and control calves were often multifocally affected similar to that observed in the trachea. The lung, tracheobronchial lymph node, tonsil, and eyelid were also evaluated and were frequently unremarkable with only rare crypt abscesses in the tonsil and scant neutrophil infiltrates in the lung.

**Figure 3:**
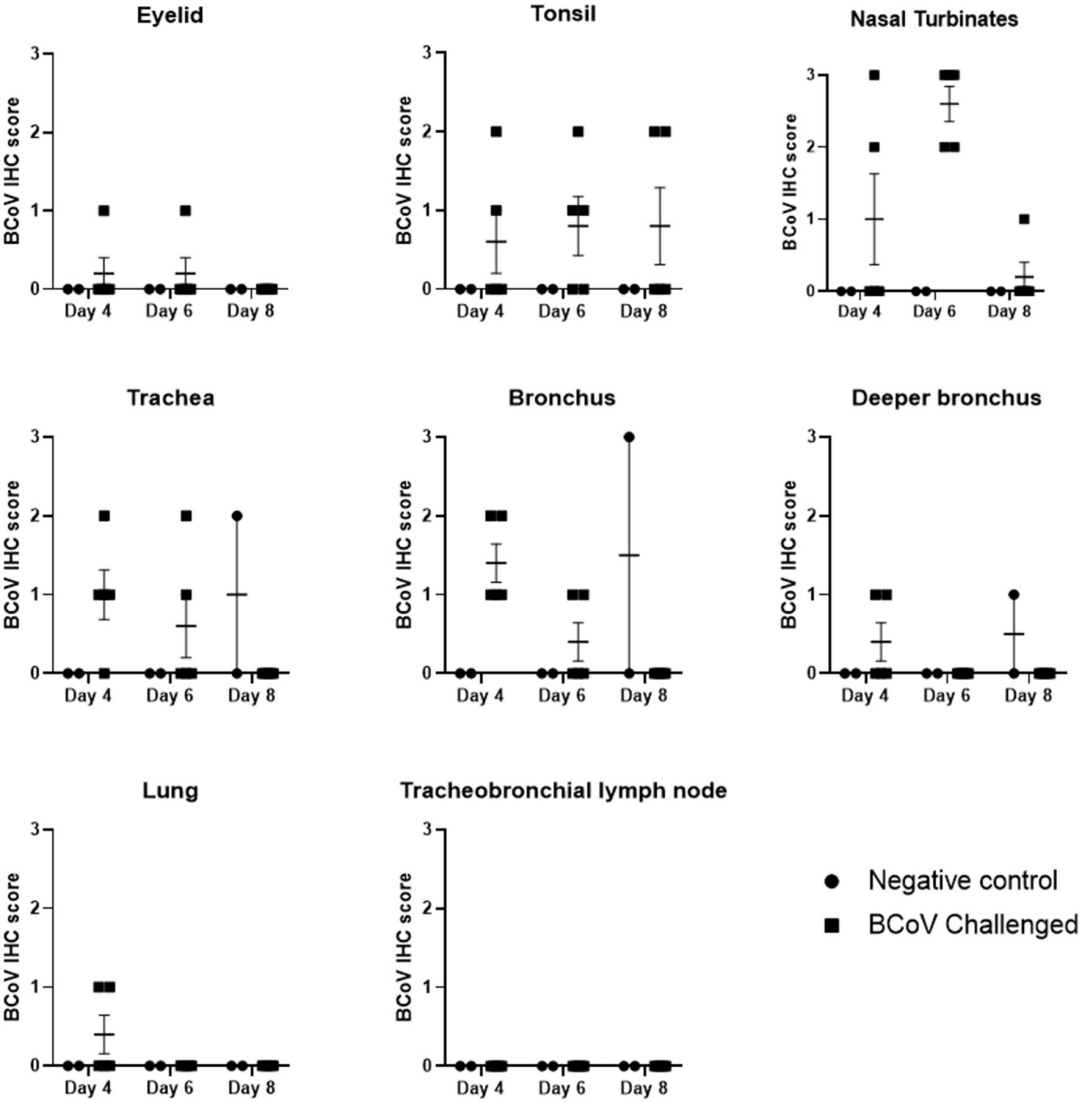
BCoV IHC scores in fixed tissues at days 4, 6, or 8 Post-challenge. Immunohistochemistry analysis of the tissues showed positive staining for BCV infection in all of the different tissue types from challenged calves, except for lymph node. The diagnostic laboratory scored the tissues as negative (0), some positive staining (1), moderate positive staining (2) or extensive positive staining (3). All of the challenged calves had moderate to extensive levels of positive staining in the nasal turbinates on day 6 Post-infection. There was a reduction in the incidence and extent of positive staining by day 8 Post-challenge, which may indicate some resolution of the BCV infection. Only one of the control calves had IHC positive tissues on day 8.

**Figure 4:**
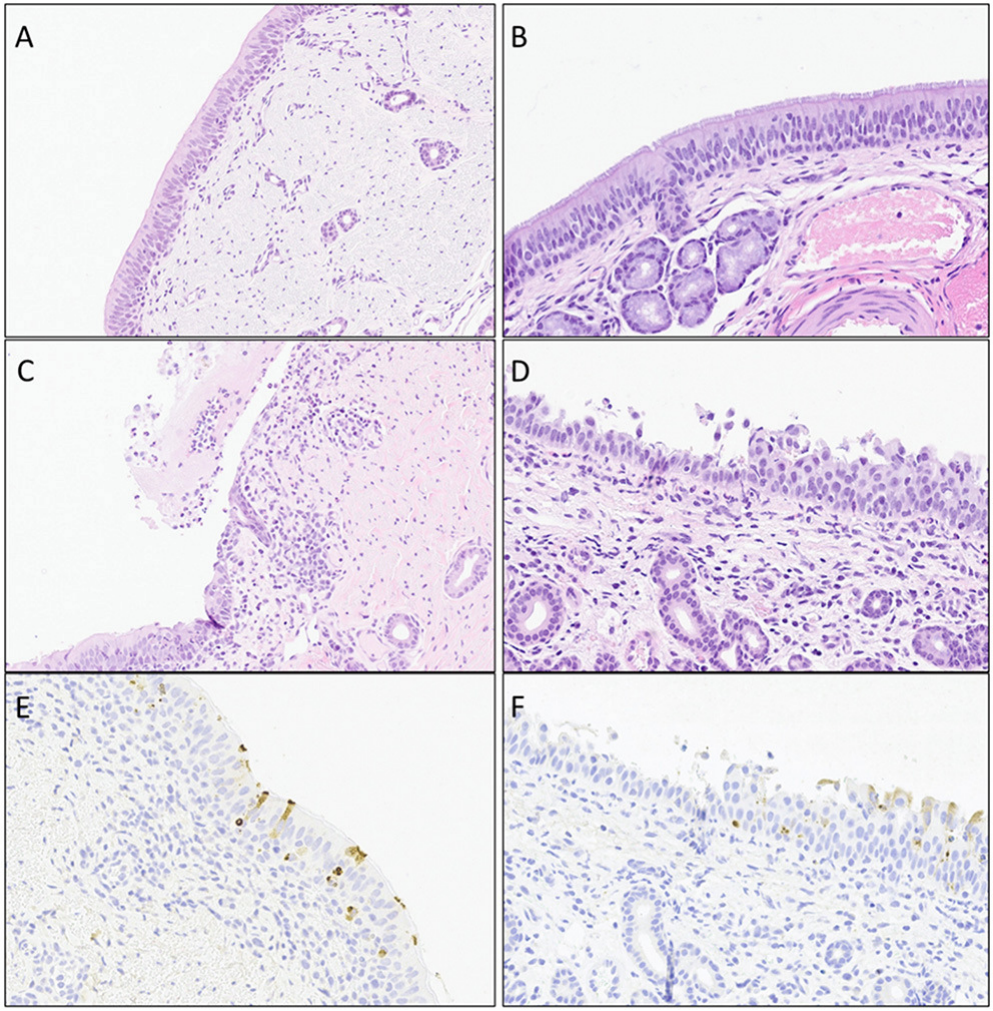
Representative images of histopathology (H&E) lesions and BCoV IHC in challenged calves. (**A** and **B**) Trachea at day 4 Post-challenge and nasal turbinates at day 6 Post-challenge from negative control calves, respectively. **(C)** Trachea on day 4 Post-challenge displaying epithelial ulceration with infiltration of the lamina propria by mixed inflammatory infiltrates. **(D)** Nasal turbinate on day 6 Post-challenge with locally extensive exfoliation and erosion of epithelium with infiltration of the lamina propria by lymphocytes and plasma cells. **(E)** Trachea on day 4 Post-challenge showing mulitfocal epithelium with intracytoplasmic detection of BCoV antigen. **(F)**. Nasal turbinate on day 6 Post-challenge with mulitfocal intact epithelium with intracytoplasmic BCoV antigen staining via IHC.

The tracheas of challenged animals showed mild IHC staining on day 4 Post-challenge which steadily declined to the point of all animals testing negative by day 8 Post-challenge. IHC revealed the nasal turbinates had frequent BCoV staining of epithelium with all challenged (Group 1) animals IHC positive at day 6 Post-challenge (Figures 3, 4F). The tonsil and bronchus had moderate IHC staining in several challenged animals at 4 and 6 days Post-challenge. The eyelid, deeper bronchus, and lung had mild staining in rare animals, and the tracheobronchial lymph node was uniformly negative across all time points. One negative control (Group 2) animal at day 8 Post-challenge was positive in the trachea, bronchus, and deeper bronchus.

## Discussion

Coronaviruses are part of a family of viruses associated with both enteric and respiratory diseases in a wide range of hosts (1, 3–8). BCoV has been well-established as a causative agent of diarrhea in cattle; however, its role as a respiratory pathogen and its contribution to the bovine respiratory disease complex is controversial (9–16, 19). In this study, fifteen calves were challenged intranasally with virulent BCoV in order to observe the clinical manifestation of the BCoV infection for up to 8 days after initial challenge, looking specifically for indication of clinical symptoms, pathology, and presence of viral infection in the respiratory tract, as compared to unchallenged control calves.

Challenged calves displayed a high incidence of clinical symptoms, both enteric and respiratory in nature, including elevated rectal temperatures, diarrhea, nasal discharge, and coughing (Supplementary Tables S1–S3). Symptoms peaked at 5 days Post-challenge with 90% of the remaining challenged calves showing clinical symptoms of disease. The most commonly reported symptoms in challenge calves were nasal discharge, coughing, and diarrhea.

Serum neutralization antibody titers to BCoV in challenge calves were beginning to rise at the end of the study (8 days Post-challenge), suggesting the development of an active humoral immune response. It is reasonable to hypothesize that the BCoV-specific antibody response would have reached a higher titer had serum collection continued past day 8 Post-challenge.

Nasal swabs were collected from calves 1 day prior to challenge and then 1 day through 8 days Post-challenge. Nasal viral shedding was detected from all challenged calves for the first 7 days Post-challenge, with 40% of remaining calves still shedding BCoV on the final day of the study (Table 1). Additionally, bronchoalveolar lavage performed 4 days after challenge also yielded viable BCoV from the lungs in all but one challenged calf (Table 3). These data suggest that BCoV is able to establish an infection and replicate in the lower respiratory tract and in the upper respiratory tract for at least 7 days after initial exposure.

**Table 3:** Average bronchoalveolar lavage titers (Log_10_ TCID_50_/mL).

**Group**	**BAL viral titer**	**Number and % BCoV positive**
Challenge	3.52	14 / 15 (93.3%)
Control	0.42	1 / 6 (16.7%)

Five randomly selected challenge calves and two negative control calves were euthanized on days 4, 6, or 8 Post-challenge each. At the time of euthanasia, tissue samples (nasal turbinate, trachea, tonsil, bronchi, lung, bronchial lymph node, and eyelid) were collected from each calf and submitted to a diagnostic laboratory for histological examination, immunohistochemistry, and quantitative PCR to determine the presence and amount of BCoV in the tissues (Tables 4, 5). The BCoV nucleic acid levels of challenged animals were particularly high (correlating to lower CT values) in the nasal turbinates, tonsil, lung, and trachea on days 4 and 6 Post-challenge (Figure 1). Six out of ten challenged calves were PCR positive in the eyelid at days 4 and 6 Post-challenge. Similarly, the tracheobronchial lymph node had high Ct counts in seven of ten calves at days 4 and 6 Post-challenge. Only the tonsil and nasal turbinates still had detectable BCoV nucleic acid by day 8 Post-challenge.

**Table 4:** Summary of qPCR results (number and percent positive).

**Group**	**Bronchioles**	**Eyelid**	**Lung**	**Lymph node**	**Nasal turbinate**	**Tonsil**	**Trachea**
Challenge	8/15 (53%)	6/15 (40%)	10/15 (67%)	7/15 (47%)	14/15 (93%)	15/15 (100%)	11/15 (73%)
Control	1/6 (17%)	0/6 (0%)	1/6 (17%)	0/6 (0%)	2/6 (33%)	1/6 (17%)	1/6 (17%)

**Table 5:** Summary of IHC results (number and percent positive).

**Group**	**Bronchioles**	**Eyelid**	**Lung**	**Lymph node**	**Nasal turbinate**	**Tonsil**	**Trachea**	**Deep bronchus**
Challenge	7/15 (47%)	2/15 (13%)	1/15 (7%)	0/15 (0%)	7/15 (47%)	7/15 (47%)	3/15 (20%)	2/15 (13%)
Control	1/6 (17%)	0/6 (0%)	0/6 (0%)	0/6 (0%)	0/6 (0%)	0/6 (0%)	1/6 (17%)	1/6 (17%)

Six negative control calves were not challenged, but were housed in a separate room in the same building as the challenge calves. On days 6 through 8 Post-challenge, one of the remaining unchallenged control calves began having positive titers for BCoV shedding in the nasal swab samples. While this calf did not have clinical symptoms through the end of the study, this same control calf was positive for BCoV nucleic acid by PCR in all evaluated tissues other than the tracheobronchial lymph node and eyelid. IHC for this calf was positive in the trachea, bronchus, and terminal bronchus evidencing active BCoV infection. The control calves were housed in the same building as the challenged calves and were likely exposed to the challenge virus a few days after the other calves were challenged despite the biosecurity measures that were in place. Control calves were always attended to the before the challenge calves with fresh PPE (Tyvek suits, gloves, boots, etc.) worn in each pen and changed between groups. However, despite all the preventative measures taken, the data suggest that a breach in biosecurity lead to the inadvertent infection of the control group. Infection prior to placement is unlikely, as the animals tested negative before entering the facility and never seroconverted. While this incidence was unintentional its occurrence displays the highly infectious nature of this virus and the ease at which it is transmissible.

In this study, calves were challenged by administering the virus intranasally, other research groups have shown viral shedding from the respiratory tract even with naturally occurring infections (20, 26–28). The detection of viral shedding in the nasal swab samples, as well as detection of BCoV in the respiratory tissue samples by PCR and IHC, from the inadvertently-infected control calf in the present study also bolsters claim that BCoV causes a respiratory infection. The histopathology results provide additional proof that beyond just infecting cells, BCoV causes microscopic pathology in the upper and lower respiratory tract. However, this data would support that the virus does not infect the lung parenchyma as has been previously reported (29). Nearly all animals in this study, control and challenged calves, displayed some levels of epithelial attenuation, necrosis, or regeneration in the bronchus and terminal bronchi (Figures 2, 4). While the majority of epithelial injury can be attributed to infection, suppored by the IHC, the BALs that were carried out on 4 days Post-challenge likely also caused damage to this epithelium and may be the cause of some histopathology lesions in the trachea and bronchi.

BCoV has been reported among the most frequently detected viruses in calves with respiratory symptoms (30, 31) and can lead to the occurrence of simultaneous infections with other pathogens. Fahkrajang et al. showed that infection with BCoV enhances the adherence of bacteria like *Pasteurella multocida* to upper and lower respiratory tract cells due to the upregulation of two major bacterial adhesion molecules (32). This increased susceptibility to additional infections can lead to severe incidence of pneumonia (33–35). It has also been shown that in some cases BCoV can cause chronic subclinical infections which could be a persistent source of vial shedding and may attribute to disease reoccurrence in the same group of cattle (10, 26, 36, 37). Evidence of antibody titers or vaccination against BCoV has also been shown to reduce the risk of respiratory disease is cattle (26, 38–40).

Previous studies have also evaluated the presence of BCoV shedding in both the respiratory and enteric tracts (26–28, 39, 41, 42). Additional studies have demonstrated high levels of cross-protection and cross-neutralization between isolates taken from enteric and respiratory BCoV infections (43, 44). Genotyping of clinical isolates from feces and nasal secretions did not show distinct genetic sub-lineages based on disease type (45, 46). Genetic clustering is more apparent on the basis of year and location of isolation, rather than disease manifestation (47). While close phylogenetic analysis shows clusters of Asian-American and European lineages (47, 48), comparison of the available whole genome sequences reveal a level of genetic similarity to the challenge virus used in this study of >98% for BCoV isolates globally.

BCoV has been shown to have remarkable antigenic similarities to the human coronavirus strain OC43 (HCoV-OC43), as well (49–51). Both the HCoV-OC43 virus and BCoV use *N-*acetyl-9-*O*-acetylneuraminic acid to attach to host cells (52). HCoV-OC43 symptoms in humans are typically synonymous with those of the common cold (53); however, HCoV-OC43 and BCoV are in the same genera of beta-coronaviruses as the SARS viruses and the MERS virus, as well as the Murine Hepatitis Virus, which is a mouse-infecting strain of coronavirus often used in the laboratory setting to study coronavirus disease mechanisms (1, 54, 55). Coronaviruses in general are the largest known RNA viruses with genomes between 27 and 31.5 kilobases (56). Due to the nature of the RNA viral genome, coronaviruses have a high mutation rate, as well as a high rate of recombination (54, 57). The high rate of mutation and recombination of the genome, along with the ability to be harbored as long-term or persistent infections in some animals, increases the risk that a viral mutant with an extended host range might arise (54). Vijgen et al. were the first to fully sequence the HCoV-OC43 genome and subsequently compare it to the BCoV genome, as well as other beta-coronaviruses. Genomic comparison revealed that HCoV-OC43 and BCoV demonstrated the highest similarity in all but one gene, with 99.6% identity on the nucleotide level (54). Based on phylogenetic analysis, they were able to trace the likely evolutionary split to as recent as 1890 (54). The similarity in the antigenic and genomic nature of BCoV to coronaviruses that are known to cause respiratory infections in humans strengthens the notion that BCoV is a respiratory pathogen in cattle, and that cattle have the potential as a zoonotic transmission source of coronavirus.

While previous studies support the claim of BCoV as a respiratory pathogen, there has been a shortage of BCoV challenge studies which effectively reproduce clinical respiratory disease and show gross and microscopic pathology. In this study, the data support that this BCoV challenge results in respiratory infections as evidenced by the isolation of BCoV in BAL fluids and positive qPCR, IHC, and histopathology findings in the lungs and other respiratory tissues, in addition to the clinical observations and viral isolation from the respiratory passage.

## Data Availability Statement

The original contributions presented in the study are included in the article/Supplementary Material, further inquiries can be directed to the corresponding author.

## Ethics Statement

The animal study was reviewed and approved by Institutional Animal Care and Use Committee of Merck Animal Health.

## Author Contributions

KS: drafted study design, performed animal challenge, collected samples (nasal swab, BAL, tissue samples), performed observations, collected and analyzed data, performed assays, and drafted manuscript. MR: performed all histopathology and immunohistochemistry, and assisted in drafting manuscript and editing. LP: assisted in drafting the study design and data analysis. CM and PS: performed animal husbandry duties, assisted in administration of challenge, and assisted in clinical observations and sample collection. CS: the attending veterinarian overseeing the study. He also assisted in administration of the challenge and performed the BAL. JK: oversaw all study activities, assisted in study design, and assisted in drafting and editing manuscript. All authors contributed to the article and approved the submitted version.

## Conflict of Interest

The authors and investigators for these studies are present or previous employees of Merck Animal Health.

## Publisher's Note

All claims expressed in this article are solely those of the authors and do not necessarily represent those of their affiliated organizations, or those of the publisher, the editors and the reviewers. Any product that may be evaluated in this article, or claim that may be made by its manufacturer, is not guaranteed or endorsed by the publisher.

## Supplementary Material

Supplementary Figure S1 Endoscopic image of bronchial bifurcation of BCV challenge calf. This is a representative image taken at the time of bronchoalveolar lavage from a calf 4 days after challenge with BCoV. A clear buildup of mucous and debris were observed, in addition to hyperemia and inflammation, on the lumen of the bronchi. Debris and mucous such as that shown in this image (white arrows) was observed in all of the challenge calves, while no such observations were made in the control, unchallenged calves.
