# Supplementary figures and images for: Bovine Coronavirus Infects the Respiratory Tract of Cattle Challenged Intranasally
Source: Front Vet Sci. 2022 Apr 29;9:878240. doi: 10.3389/fvets.2022.878240 (PMC9100586; doi:10.3389/fvets.2022.878240)

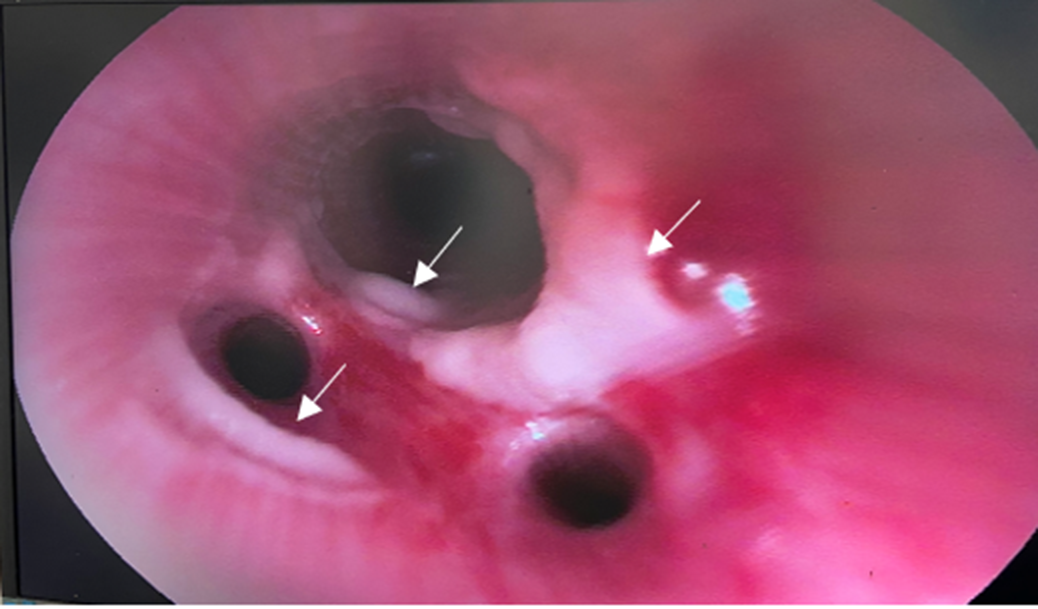

Supplement: Supplementary Figure S1 — Endoscopic image of bronchial bifurcation of BCV challenge calf. This is a representative image taken at the time of bronchoalveolar lavage from a calf 4 days after challenge with BCoV. A clear buildup of mucous and debris were observed, in addition to hyperemia and inflammation, on the lumen of the bronchi. Debris and mucous such as that shown in this image (white arrows) was observed in all of the challenge calves, while no such observations were made in the control, unchallenged calves. [file Image_1.TIF]
