# Supplementary material for: Bovine Coronavirus Infects the Respiratory Tract of Cattle Challenged Intranasally
Source: Front Vet Sci. 2022 Apr 29;9:878240. doi: 10.3389/fvets.2022.878240 (PMC9100586; doi:10.3389/fvets.2022.878240)
Supplement: Supplementary file 2 [file Table_1.docx]

**Supplemental Table S1. Clinical Observations**

|  | | | Day -3 | Day -2 | Day -1 | Challenge | Day 1 | Day 2 | Day 3 | Day 4 | Day 5 | Day 6 | Day 7 | Day 8 |
| --- | --- | --- | --- | --- | --- | --- | --- | --- | --- | --- | --- | --- | --- | --- |
|  | Calf # | ChalPen | 19-Oct-20 | 20-Oct-20 | 21-Oct-20 | 22-Oct-20 | 23-Oct-20 | 24-Oct-20 | 25-Oct-20 | 26-Oct-20 | 27-Oct-20 | 28-Oct-20 | 29-Oct-20 | 30-Oct-20 |
|  | Group 1A (necropsy day 4) | | | |  |  |  |  |  |  |  |  |  |  |
|  | 669 | 111 | 0 | 0 | 0 | 0 | 0 | 0 | 0 | N2 |  |  |  |  |
|  | 678 | 112 | 0 | 0 | 0 | 0 | 0 | 0 | 0 | 0 |  |  |  |  |
|  | 683 | 111 | 0 | 0 | 0 | 0 | 0 | R | 0 | N1 |  |  |  |  |
|  | 685 | 110 | NA* | NA* | 0 | 0 | 0 | 0 | 0 | 0 |  |  |  |  |
|  | 686 | 110 | 0 | 0 | 0 | 0 | 0 | 0 | 0 | 0 |  |  |  |  |
| Group 1B (necropsy day 6) | | | |  |  |  |  |  |  |  |  |  |  |  |
|  | 672 | 110 | 0 | 0 | 0 | 0 | 0 | 0 | 0 | F, N1 | F, R | 0 |  |  |
|  | 673 | 110 | 0 | 0 | 0 | 0 | 0 | R, D2 | 0 | N1 | N2 | 0 |  |  |
|  | 675 | 111 | 0 | 0 | 0 | 0 | 0 | D1 | 0 | 0 | N1 | 0 |  |  |
|  | 679 | 112 | 0 | 0 | 0 | 0 | 0 | 0 | 0 | N1 | N2 | C1 |  |  |
|  | 687 | 112 | 0 | 0 | 0 | 0 | F | D1 | 0 | N2 | N2, C1 | N2, C1 |  |  |
| Group 1C (necropsy day 8) | | | |  |  |  |  |  |  |  |  |  |  |  |
|  | 668 | 112 | 0 | 0 | 0 | 0 | 0 | 0 | 0 | 0 | 0 | C1 | N2, C1 | 0 |
|  | 671 | 112 | 0 | 0 | 0 | 0 | 0 | D1 | 0 | F | N1, C1 | N2, D1, C1 | N2, C1 | 0 |
|  | 674 | 111 | 0 | 0 | 0 | 0 | 0 | R, D1 | F | N1 | N1, D2 | N2, C1 | N2, D2 | 0 |
|  | 681 | 110 | 0 | 0 | 0 | 0 | 0 | 0 | 0 | 0 | F, N1 | D1, C1 | 0 | 0 |
|  | 689 | 111 | 0 | 0 | 0 | 0 | 0 | D1 | 0 | F, N2, D2 | N1 | 0 | N2 | 0 |
|  |  |  |  |  |  |  |  |  |  |  |  |  |  |  |
|  | | | |  |  |  |  |  |  |  |  |  |  |  |
|  |  |  |  |  |  |  |  |  |  |  |  |  |  |  |
|  | Calf # | ChalPen | 19-Oct-20 | 20-Oct-20 | 21-Oct-20 | 22-Oct-20 | 23-Oct-20 | 24-Oct-20 | 25-Oct-20 | 26-Oct-20 | 27-Oct-20 | 28-Oct-20 | 29-Oct-20 | 30-Oct-20 |
|  | Group 2A (necropsy day 4) | | |  |  |  |  |  |  |  |  |  |  |  |
|  | 680 | 113 | 0 | 0 | N1 | 0 | 0 | 0 | F | 0 |  |  |  |  |
|  | 682 | 113 | 0 | 0 | 0 | 0 | 0 | 0 | 0 | D2 |  |  |  |  |
| Group 2B (necropsy day 6) | | | |  |  |  |  |  |  |  |  |  |  |  |
|  | 688 | 113 | 0 | 0 | 0 | 0 | 0 | 0 | F | 0 | 0 | D2 |  |  |
|  | 690 | 113 | 0 | 0 | 0 | 0 | 0 | 0 | 0 | 0 | 0 | 0 |  |  |
| Group 2C (necropsy day 8) | | | |  |  |  |  |  |  |  |  |  |  |  |
|  | 684 | 113 | 0 | 0 | 0 | 0 | 0 | 0 | 0 | 0 | 0 | 0 | 0 | 0 |
|  | 691 | 113 | 0 | 0 | 0 | 0 | 0 | 0 | 0 | 0 | 0 | 0 | 0 | 0 |
|  |  |  |  |  |  |  |  |  |  |  |  |  |  |  |
|  |  |  | | | | | | | |  |  |  |  |  |

F = fever ≥103.0 °F

R = respiratory rate ≥60 breaths/minute

N = nasal discharge (1 = mild, small amount of unilateral slightly cloudy mucus; 2 = moderate; bilateral cloudy mucus discharge; 3 = severe; copious bilateral purulent discharge)

D = diarrhea (1 = Mild; semi-formed feces, pasty; 2 = Moderate, loose feces with or without blood, fibrin, or pseudomembrane; 3 = severe, watery feces, may be projectile, with or without blood, fibrin, or pseudomembrane)

C = cough (1 = single cough, induced; 2 = occasional spontaneous cough or repeated induced cough; 3 = repeated spontaneous cough)

*Calf #685 replaced another calf that was excluded from the study on Day -1, thus there was no clinical observation data recorded for Calf #685 on Day -2 or -3.

|  | Calf # | ChalPen | 19-Oct-20 | 20-Oct-20 | 21-Oct-20 | 22-Oct-20 | 23-Oct-20 | 24-Oct-20 | 25-Oct-20 | 26-Oct-20 | 27-Oct-20 | 28-Oct-20 | 29-Oct-20 | 30-Oct-20 |
| --- | --- | --- | --- | --- | --- | --- | --- | --- | --- | --- | --- | --- | --- | --- |
| Group 1A (necropsy day 4) | | | |  |  |  |  |  |  |  |  |  |  |  |
|  | 669 | 111 | 102.4 | 101.7 | 101.8 | 101.8 | 101.5 | 101.4 | 102.6 | 102.6 |  |  |  |  |
|  | 678 | 112 | 102.4 | 102.4 | 102.1 | 102.1 | 101.8 | 102.1 | 102.7 | 102.7 |  |  |  |  |
|  | 683 | 111 | 101.7 | 101.5 | 101.4 | 102.4 | 101.9 | 102.5 | 102.3 | 101.2 |  |  |  |  |
|  | 685 | 110 | NA* | NA* | 101.1 | 101.1 | 101.9 | 101.7 | 102.1 | 101.4 |  |  |  |  |
|  | 686 | 110 | 102.0 | 101.2 | 102.8 | 101.3 | 102.8 | 102.4 | 101.8 | 102.0 |  |  |  |  |
| Group 1B (necropsy day 6) | | | |  |  |  |  |  |  |  |  |  |  |  |
|  | 672 | 110 | 101.9 | 101.7 | 102.8 | 102.9 | 102.0 | 102.7 | 102.3 | **103.6** | **103.1** | 101.8 |  |  |
|  | 673 | 110 | 102.3 | 102.4 | 102.5 | 102.3 | 102.5 | 102.1 | 102.5 | 102.7 | 102.6 | 101.9 |  |  |
|  | 675 | 111 | 101.3 | 102.1 | 102.3 | 102.2 | 101.9 | 101.9 | 101.7 | 101.5 | 102.0 | 101.4 |  |  |
|  | 679 | 112 | 101.6 | 100.9 | 101.5 | 102.0 | 101.7 | 102.5 | 102.1 | 101.1 | 102.0 | 101.0 |  |  |
|  | 687 | 112 | 102.1 | 102.1 | 102.8 | 102.3 | **103.0** | 102.3 | 102.3 | 102.0 | 101.2 | 101.1 |  |  |
| Group 1C (necropsy day 8) | | | |  |  |  |  |  |  |  |  |  |  |  |
|  | 668 | 112 | 101.4 | 101.3 | 101.7 | 101.4 | 101.9 | 102.1 | 101.5 | 102.5 | 102.1 | 101.3 | 101.0 | 101.6 |
|  | 671 | 112 | 101.8 | 101.0 | 102.1 | 101.9 | 102.2 | 102.6 | 101.2 | **103.0** | 102.4 | 101.9 | 101.2 | 101.5 |
|  | 674 | 111 | 102.2 | 101.2 | 102.3 | 101.0 | 101.5 | 102.8 | **103.0** | 102.6 | 101.5 | 101.3 | 101.1 | 102.0 |
|  | 681 | 110 | 101.4 | 101.8 | 102.1 | 102.9 | 102.6 | 101.9 | 101.8 | 102.0 | **103.0** | 103.0 | 102.2 | 102.3 |
|  | 689 | 111 | 101.2 | 101.8 | 101.4 | 102.0 | 102.3 | 102.3 | 101.9 | **103.0** | 102.6 | 102.0 | 101.4 | 102.8 |
|  | **Average** |  | **101.8** | **101.7** | **102.0** | **102.0** | **102.1** | **102.2** | **102.1** | **102.3** | **102.3** | **101.7** | **101.4** | **102.0** |
|  |  |  |  |  |  |  |  |  |  |  |  |  |  |  |
|  |  |  |  |  |  |  |  |  |  |  |  |  |  |  |
|  | Calf # | ChalPen | 19-Oct-20 | 20-Oct-20 | 21-Oct-20 | 22-Oct-20 | 23-Oct-20 | 24-Oct-20 | 25-Oct-20 | 26-Oct-20 | 27-Oct-20 | 28-Oct-20 | 29-Oct-20 | 30-Oct-20 |
| Group 2A (necropsy day 4) | | | |  |  |  |  |  |  |  |  |  |  |  |
|  | 680 | 113 | 101.9 | 101.8 | 102.5 | 102.7 | 101.2 | 102.2 | **103.2** | 101.2 |  |  |  |  |
|  | 682 | 113 | 102.0 | 101.3 | 101.9 | 102.5 | 101.8 | 101.8 | 101.5 | 101.9 |  |  |  |  |
| Group 2B (necropsy day 6) | | | |  |  |  |  |  |  |  |  |  |  |  |
|  | 688 | 113 | 101.3 | 101.2 | 101.3 | 102.1 | 101.1 | 102.1 | **103.1** | 101.6 | 101.3 | 101.1 |  |  |
|  | 690 | 113 | 102.4 | 101.6 | 101.9 | 102.6 | 102.0 | 102.3 | 102.0 | 101.7 | 101.0 | 101.3 |  |  |
| Group 2C (necropsy day 8) | | | |  |  |  |  |  |  |  |  |  |  |  |
|  | 684 | 113 | 102.2 | 100.9 | 101.9 | 102.3 | 101.5 | 101.5 | 102.5 | 101.3 | 101.1 | 101.0 | 101.2 | 101.5 |
|  | 691 | 113 | 102.7 | 101.1 | 101.1 | 101.5 | 101.2 | 101.6 | 102.2 | 101.1 | 101.2 | 101.6 | 101.1 | 101.9 |
|  | **Average** |  | **102.1** | **101.3** | **101.8** | **102.3** | **101.5** | **101.9** | **102.4** | **101.5** | **101.2** | **101.3** | **101.2** | **101.7** |

**Supplemental Table S2. Rectal Temperatures**

*Calf #685 replaced another calf that was excluded from the study on Day -1, thus there was no clinical observation data recorded for Calf #685 on Day -2 or -3.

**Supplemental Table S3. Respiratory Rates (Breaths/Minute)**

|  |  |  | Day -3 | Day -2 | Day -1 | Challenge | Day 1 | Day 2 | Day 3 | Day 4 | Day 5 | Day 6 | Day 7 | Day 8 |
| --- | --- | --- | --- | --- | --- | --- | --- | --- | --- | --- | --- | --- | --- | --- |
|  | Calf # | ChalPen | 19-Oct-20 | 20-Oct-20 | 21-Oct-20 | 22-Oct-20 | 23-Oct-20 | 24-Oct-20 | 25-Oct-20 | 26-Oct-20 | 27-Oct-20 | 28-Oct-20 | 29-Oct-20 | 30-Oct-20 |
| Group 1A (necropsy day 4) | | | |  |  |  |  |  |  |  |  |  |  |  |
|  | 669 | 111 | 32 | 40 | 32 | 36 | 36 | 44 | 36 | 36 |  |  |  |  |
|  | 678 | 112 | 40 | 36 | 40 | 40 | 36 | 36 | 32 | 36 |  |  |  |  |
|  | 683 | 111 | 28 | 48 | 36 | 32 | 40 | **80** | 40 | 28 |  |  |  |  |
|  | 685 | 110 | NA* | NA* | 52 | 40 | 52 | 48 | 48 | 40 |  |  |  |  |
|  | 686 | 110 | 40 | 32 | 48 | 36 | 36 | 36 | 36 | 40 |  |  |  |  |
| Group 1B (necropsy day 6) | | | |  |  |  |  |  |  |  |  |  |  |  |
|  | 672 | 110 | 28 | 32 | 40 | 40 | 36 | 36 | 40 | 32 | **60** | 28 |  |  |
|  | 673 | 110 | 36 | 36 | 40 | 40 | 44 | 36 | 32 | 36 | 36 | 32 |  |  |
|  | 675 | 111 | 28 | 40 | 32 | 32 | 40 | 36 | 36 | 44 | 36 | 28 |  |  |
|  | 679 | 112 | 32 | 44 | 48 | 36 | 32 | **68** | 36 | 40 | 40 | 32 |  |  |
|  | 687 | 112 | 36 | 36 | 36 | 36 | 36 | 36 | 40 | 36 | 32 | 32 |  |  |
| Group 1C (necropsy day 8) | | | |  |  |  |  |  |  |  |  |  |  |  |
|  | 668 | 112 | 36 | 40 | 32 | 36 | 40 | 36 | 40 | 36 | 28 | 28 | 24 | 35 |
|  | 671 | 112 | 28 | 36 | 40 | 36 | 40 | 36 | 40 | 36 | 32 | 36 | 28 | 24 |
|  | 674 | 111 | 28 | 44 | 40 | 32 | 40 | **72** | 36 | 32 | 36 | 32 | 36 | 20 |
|  | 681 | 110 | 32 | 36 | 32 | 28 | 36 | 48 | 36 | 32 | 32 | 28 | 28 | 24 |
|  | 689 | 111 | 32 | 44 | 36 | 28 | 40 | 52 | 52 | 28 | 36 | 28 | 32 | 35 |
|  | **Average** |  | **32.6** | **38.9** | **38.9** | **35.2** | **38.9** | **46.7** | **38.7** | **35.5** | **36.8** | **30.4** | **29.6** | **27.6** |
|  |  |  |  |  |  |  |  |  |  |  |  |  |  |  |
|  |  |  |  |  |  |  |  |  |  |  |  |  |  |  |
|  | Calf # | ChalPen | 19-Oct-20 | 20-Oct-20 | 21-Oct-20 | 22-Oct-20 | 23-Oct-20 | 24-Oct-20 | 25-Oct-20 | 26-Oct-20 | 27-Oct-20 | 28-Oct-20 | 29-Oct-20 | 30-Oct-20 |
| Group 2A (necropsy day 4) | | | |  |  |  |  |  |  |  |  |  |  |  |
|  | 680 | 113 | 40 | 40 | 40 | 52 | 40 | 36 | 32 | 32 |  |  |  |  |
|  | 682 | 113 | 36 | 48 | 36 | 40 | 28 | 40 | 40 | 32 |  |  |  |  |
| Group 2B (necropsy day 6) | | | |  |  |  |  |  |  |  |  |  |  |  |
|  | 688 | 113 | 32 | 56 | 44 | 36 | 40 | 52 | 28 | 28 | 40 | 40 |  |  |
|  | 690 | 113 | 32 | 44 | 44 | 44 | 36 | 40 | 36 | 28 | 32 | 28 |  |  |
| Group 2C (necropsy day 8) | | | |  |  |  |  |  |  |  |  |  |  |  |
|  | 684 | 113 | 44 | 48 | 40 | 32 | 36 | 52 | 32 | 32 | 32 | 32 | 24 | 24 |
|  | 691 | 113 | 36 | 32 | 40 | 36 | 52 | 40 | 28 | 28 | 52 | 36 | 28 | 20 |
|  | **Average** |  | **36.7** | **44.7** | **40.7** | **40.0** | **38.7** | **43.3** | **32.7** | **30.0** | **39.0** | **34.0** | **26.0** | **22.0** |

*Calf #685 replaced another calf that was excluded from the study on Day -1, thus there was no clinical observation data recorded for Calf #685 on Day -2 or -3.

**Supplemental Table S4. Nasal swab virus shedding (Log_10_ TCID_50_/mL)**

|  |  |  | Day -1 | Day 1 | Day 2 | Day 3 | Day 4 | Day 5 | Day 6 | Day 7 | Day 8 |
| --- | --- | --- | --- | --- | --- | --- | --- | --- | --- | --- | --- |
|  | Calf # | ChalPen | 21-Oct-20 | 23-Oct-20 | 24-Oct-20 | 25-Oct-20 | 26-Oct-20 | 27-Oct-20 | 28-Oct-20 | 29-Oct-20 | 30-Oct-20 |
| Group 1A (necropsy day 4) | | |  |  |  |  |  |  |  |  |  |
|  | 669 | 111 | 0.0 | 2.5 | 4.8 | 8.3 | 6.3 |  |  |  |  |
|  | 678 | 112 | 0.0 | 5.5 | 7.0 | 6.0 | 6.3 |  |  |  |  |
|  | 683 | 111 | 0.0 | 4.8 | 5.5 | 6.3 | 5.0 |  |  |  |  |
|  | 685 | 110 | 0.0 | 4.5 | 4.0 | 5.3 | 4.5 |  |  |  |  |
|  | 686 | 110 | 0.0 | 3.5 | 5.8 | 6.8 | 5.3 |  |  |  |  |
| Group 1B (necropsy day 6) | | | 0.0 |  |  |  |  |  |  |  |  |
|  | 672 | 110 | 0.0 | 4.0 | 8.8 | 6.8 | 7.8 | 6.5 | 5.5 |  |  |
|  | 673 | 110 | 0.0 | 3.0 | 6.3 | 6.3 | 8.3 | 7.8 | 5.3 |  |  |
|  | 675 | 111 | 0.0 | 2.8 | 6.3 | 5.0 | 5.5 | 7.0 | 5.8 |  |  |
|  | 679 | 112 | 0.0 | 4.0 | 7.0 | 9.5 | 6.8 | 5.0 | 4.5 |  |  |
|  | 687 | 112 | 0.0 | 4.8 | 9.0 | 6.0 | 5.5 | 6.3 | 5.5 |  |  |
| Group 1C (necropsy day 8) | | | 0.0 |  |  |  |  |  |  |  |  |
|  | 668 | 112 | 0.0 | 4.0 | 5.0 | 5.3 | 5.0 | 8.0 | 5.0 | 5.3 | 5.0 |
|  | 671 | 112 | 0.0 | 0.0 | 6.5 | 5.5 | 5.8 | 8.8 | 4.0 | 4.3 | 0.0 |
|  | 674 | 111 | 0.0 | 0.0 | 3.8 | 4.3 | 5.0 | 4.3 | 9.0 | 4.0 | 2.0 |
|  | 681 | 110 | 0.0 | 8.0 | 6.5 | 6.3 | 8.5 | 6.3 | 4.0 | 2.0 | 0.0 |
|  | 689 | 111 | 0.0 | 6.0 | 6.8 | 8.3 | 5.3 | 6.0 | 6.0 | 9.3 | 0.0 |
|  | **Average** |  | **0.0** | **3.7** | **6.2** | **6.4** | **6.0** | **6.6** | **5.5** | **4.0** | **1.4** |
|  |  |  |  |  |  |  |  |  |  |  |  |
|  |  |  |  |  |  |  |  |  |  |  |  |
|  | Calf # | ChalPen | 21-Oct-20 | 23-Oct-20 | 24-Oct-20 | 25-Oct-20 | 26-Oct-20 | 27-Oct-20 | 28-Oct-20 | 29-Oct-20 | 30-Oct-20 |
| Group 2A (necropsy day 4) | | |  |  |  |  |  |  |  |  |  |
|  | 680 | 113 | 0.0 | 0.0 | 0.0 | 0.0 | 0.0 |  |  |  |  |
|  | 682 | 113 | 0.0 | 0.0 | 0.0 | 0.0 | 0.0 |  |  |  |  |
| Group 2B (necropsy day 6) | | | 0.0 |  |  |  |  |  |  |  |  |
|  | 688 | 113 | 0.0 | 0.0 | 0.0 | 0.0 | 0.0 | 0.0 | 0.0 |  |  |
|  | 690 | 113 | 0.0 | 0.0 | 0.0 | 0.0 | 0.0 | 0.0 | 0.0 |  |  |
| Group 2C (necropsy day 8) | | | 0.0 |  |  |  |  |  |  |  |  |
|  | 684 | 113 | 0.0 | 0.0 | 0.0 | 0.0 | 0.0 | 0.0 | 3.3 | 9.3 | 9.5 |
|  | 691 | 113 | 0.0 | 0.0 | 0.0 | 0.0 | 0.0 | 0.0 | 0.0 | 0.0 | 2.0 |
|  | **Average** |  | **0.0** | **0.0** | **0.0** | **0.0** | **0.0** | **0.0** | **0.8** | **4.6** | **5.8** |

**Supplemental Table S5. Serum neutralization antibody titers**

| Group 1A (necropsy day 4) | | | Day -8 | Day 4 | Day 6 | Day 8 |
| --- | --- | --- | --- | --- | --- | --- |
|  | MAH # | ChalPen | 14-Oct-20 | 26-Oct-20 | 28-Oct-20 | 30-Oct-20 |
|  | 669 | 111 | 64 | 64 |  |  |
|  | 678 | 112 | 64 | 32 |  |  |
|  | 683 | 111 | 32 | 32 |  |  |
|  | 685 | 110 | 32 | 64 |  |  |
|  | 686 | 110 | 32 | 32 |  |  |
| Group 1B (necropsy day 6) | | |  |  |  |  |
|  | 672 | 110 | 32 |  | 32 |  |
|  | 673 | 110 | 64 |  | 64 |  |
|  | 675 | 111 | 32 |  | 64 |  |
|  | 679 | 112 | 32 |  | 32 |  |
|  | 687 | 112 | 64 |  | 64 |  |
| Group 1C (necropsy day 8) | | |  |  |  |  |
|  | 668 | 112 | 32 |  |  | 64 |
|  | 671 | 112 | 32 |  |  | 64 |
|  | 674 | 111 | 32 |  |  | 128 |
|  | 681 | 110 | 64 |  |  | 128 |
|  | 689 | 111 | 64 |  |  | 128 |
|  | **GMT** |  | **41.0** | **42.2** | **48.5** | **90.5** |
|  |  |  |  |  |  |  |
| Group 2A (necropsy day 4) | | |  |  |  |  |
|  | MAH # | ChalPen | 14-Oct-20 | 26-Oct-20 | 28-Oct-20 | 30-Oct-20 |
|  | 680 | 113 | 32 | 64 |  |  |
|  | 682 | 113 | 64 | 32 |  |  |
| Group 2B (necropsy day 6) | | |  |  |  |  |
|  | 688 | 113 | 64 |  | 32 |  |
|  | 690 | 113 | 64 |  | 32 |  |
| Group 2C (necropsy day 8) | | |  |  |  |  |
|  | 684 | 113 | 64 |  |  | 64 |
|  | 691 | 113 | 64 |  |  | 32 |
|  | **GMT** |  | **57.0** | **45.3** | **32.0** | **45.3** |
|  |  |  |  |  |  |  |

*Any sample with an antibody titer less than the cutoff of 64 was assigned a value of (and was recorded as) 32 for determination of GMT

**Supplemental Table S6. RT-rtPCR results**

| Calf # | ChalPen | Bronchioles | | Eyelid | | Lung | | Lymph Node | | Nasal Turbinate | Tonsil | Trachea |
| --- | --- | --- | --- | --- | --- | --- | --- | --- | --- | --- | --- | --- |
|  |  |  |  | |  | |  | |  |  |  |  |
| 669 | 111 | + | | 0 | | + | | + | | + | + | + |
| 678 | 112 | + | | + | | + | | + | | + | + | + |
| 683 | 111 | + | | + | | + | | + | | + | + | + |
| 685 | 110 | + | | + | | + | | + | | + | + | + |
| 686 | 110 | + | | 0 | | 0 | | 0 | | + | + | + |
|  |  |  |  | |  | |  | |  |  |  |  |
| 672 | 110 | 0 | | 0 | | 0 | | 0 | | + | + | + |
| 673 | 110 | 0 | | 0 | | + | | 0 | | + | + | + |
| 675 | 111 | + | | + | | + | | + | | + | + | 0 |
| 679 | 112 | + | | + | | + | | + | | + | + | + |
| 687 | 112 | + | | + | | + | | + | | + | + | + |
|  |  |  |  | |  | |  | |  |  |  |  |
| 668 | 112 | 0 | | 0 | | 0 | | 0 | | + | + | 0 |
| 671 | 112 | 0 | | 0 | | + | | 0 | | 0 | + | 0 |
| 674 | 111 | 0 | | 0 | | + | | 0 | | + | + | + |
| 681 | 110 | 0 | | 0 | | 0 | | 0 | | + | + | + |
| 689 | 111 | 0 | | 0 | | 0 | | 0 | | + | + | 0 |
|  |  |  | |  | |  | |  | |  |  |  |
|  |  |  | |  | |  | |  | |  |  |  |
| Calf # | ChalPen |  | |  | |  | |  | |  |  |  |
|  |  |  |  | |  | |  | |  |  |  |  |
| 680 | 113 | 0 | | 0 | | 0 | | 0 | | 0 | 0 | 0 |
| 682 | 113 | 0 | | 0 | | 0 | | 0 | | 0 | 0 | 0 |
|  |  |  |  | |  | |  | |  |  |  |  |
| 688 | 113 | 0 | | 0 | | 0 | | 0 | | 0 | 0 | 0 |
| 690 | 113 | 0 | | 0 | | 0 | | 0 | | 0 | 0 | 0 |
|  |  |  |  | |  | |  | |  |  |  |  |
| 684 | 113 | + | | 0 | | 0 | | 0 | | + | + | + |
| 691 | 113 | 0 | | 0 | | + | | 0 | | + | 0 | 0 |
|  |  |  | |  | |  | |  | |  |  |  |

+ = positive PCR result for BCoV; 0 = negative PCR result for BCoV

**Supplemental Table S7. RT-rtPCR CT values**

| Calf # | ChalPen | Bronchioles | | Eyelid | | Lung | | Lymph Node | | Nasal Turbinate | Tonsil | Trachea |
| --- | --- | --- | --- | --- | --- | --- | --- | --- | --- | --- | --- | --- |
|  |  |  |  | |  | |  | |  |  |  |  |
| 669 | 111 | 26.4 | | ≥35 | | 26.8 | | 34.7 | | 23.6 | 25.7 | 24.6 |
| 678 | 112 | 30.2 | | 28.5 | | 33.6 | | 34.6 | | 28.1 | 31.3 | 27.4 |
| 683 | 111 | 30.4 | | 32.4 | | 33.3 | | 34.2 | | 30.1 | 27.7 | 30.6 |
| 685 | 110 | 26.6 | | 31.6 | | 32.0 | | 31.2 | | 27.0 | 25.7 | 32.6 |
| 686 | 110 | 32.6 | | ≥35 | | ≥35 | | ≥35 | | 31.6 | 27.7 | 28.8 |
|  |  |  |  | |  | |  | |  |  |  |  |
| 672 | 110 | ≥35 | | ≥35 | | ≥35 | | ≥35 | | 24.3 | 30.2 | 30.9 |
| 673 | 110 | ≥35 | | ≥35 | | 30.4 | | ≥35 | | 25.0 | 27.1 | 30.6 |
| 675 | 111 | 32.2 | | 28.8 | | 29.0 | | 31.8 | | 23.7 | 28.3 | ≥35 |
| 679 | 112 | 32.2 | | 34.7 | | 30.0 | | 33.9 | | 24.0 | 31.4 | 27.1 |
| 687 | 112 | 29.0 | | 31.3 | | 29.2 | | 31.1 | | 27.3 | 30.9 | 26.2 |
|  |  |  |  | |  | |  | |  |  |  |  |
| 668 | 112 | ≥35 | | ≥35 | | ≥35 | | ≥35 | | 31.7 | 30.8 | ≥35 |
| 671 | 112 | ≥35 | | ≥35 | | 34.3 | | ≥35 | | ≥35 | 31.6 | ≥35 |
| 674 | 111 | ≥35 | | ≥35 | | 34.2 | | ≥35 | | 32.5 | 34.1 | 34.8 |
| 681 | 110 | ≥35 | | ≥35 | | ≥35 | | ≥35 | | 32.9 | 34.6 | 24.3 |
| 689 | 111 | ≥35 | | ≥35 | | ≥35 | | ≥35 | | 26.9 | 30.9 | ≥35 |
|  |  |  | |  | |  | |  | |  |  |  |
|  |  |  | |  | |  | |  | |  |  |  |
| Calf # | ChalPen |  | |  | |  | |  | |  |  |  |
|  |  |  |  | |  | |  | |  |  |  |  |
| 680 | 113 | ≥35 | | ≥35 | | ≥35 | | ≥35 | | ≥35 | ≥35 | ≥35 |
| 682 | 113 | ≥35 | | ≥35 | | ≥35 | | ≥35 | | ≥35 | ≥35 | ≥35 |
|  |  |  |  | |  | |  | |  |  |  |  |
| 688 | 113 | ≥35 | | ≥35 | | ≥35 | | ≥35 | | ≥35 | ≥35 | ≥35 |
| 690 | 113 | ≥35 | | ≥35 | | ≥35 | | ≥35 | | ≥35 | ≥35 | ≥35 |
|  |  |  |  | |  | |  | |  |  |  |  |
| 684 | 113 | 24.3 | | ≥35 | | ≥35 | | ≥35 | | 26.9 | 30.9 | 25.2 |
| 691 | 113 | ≥35 | | ≥35 | | 34.9 | | ≥35 | | 34.3 | ≥35 | ≥35 |
|  |  |  | |  | |  | |  | |  |  |  |

**Supplemental Table S8. Tissue sample immunohistochemistry scores**

| Calf # | ChalPen | Bronchioles | Eyelid | Lung | Lymph Node | Nasal Turbinate | Tonsil | Trachea | Deep Bronchus |
| --- | --- | --- | --- | --- | --- | --- | --- | --- | --- |
|  |  |  |  |  |  |  |  |  |  |
| 669 | 111 | 2 | 0 | 1 | 0 | 0 | 0 | 0 | 1 |
| 678 | 112 | 1 | 0 | 1 | 0 | 3 | 0 | 1 | 0 |
| 683 | 111 | 1 | 1 | 0 | 0 | 0 | 1 | 0 | 0 |
| 685 | 110 | 2 | 0 | 0 | 0 | 0 | 0 | 0 | 1 |
| 686 | 110 | 1 | 0 | 0 | 0 | 0 | 2 | 1 | 0 |
|  |  |  |  |  |  |  |  |  |  |
| 672 | 110 | 0 | 0 | 0 | 0 | 3 | 0 | 0 | 0 |
| 673 | 110 | 0 | 0 | 0 | 0 | 3 | 0 | 0 | 0 |
| 675 | 111 | 0 | 1 | 0 | 0 | 2 | 2 | 0 | 0 |
| 679 | 112 | 1 | 0 | 0 | 0 | 3 | 1 | 2 | 0 |
| 687 | 112 | 1 | 0 | 0 | 0 | 2 | 1 | 0 | 0 |
|  |  |  |  |  |  |  |  |  |  |
| 668 | 112 | 0 | 0 | 0 | 0 | 1 | 0 | 0 | 0 |
| 671 | 112 | 0 | 0 | 0 | 0 | 0 | 2 | 0 | 0 |
| 674 | 111 | 0 | 0 | 0 | 0 | 0 | 0 | 0 | 0 |
| 681 | 110 | 0 | 0 | 0 | 0 | 0 | 0 | 0 | 0 |
| 689 | 111 | 0 | 0 | 0 | 0 | 0 | 2 | 0 | 0 |
|  |  |  |  |  |  |  |  |  |  |
| Calf # | ChalPen |  |  |  |  |  |  |  |  |
|  |  |  |  |  |  |  |  |  |  |
| 680 | 113 | 0 | 0 | 0 | 0 | 0 | 0 | 0 | 0 |
| 682 | 113 | 0 | 0 | 0 | 0 | 0 | 0 | 0 | 0 |
|  |  |  |  |  |  |  |  |  |  |
| 688 | 113 | 0 | 0 | 0 | 0 | 0 | 0 | 0 | 0 |
| 690 | 113 | 0 | 0 | 0 | 0 | 0 | 0 | 0 | 0 |
|  |  |  |  |  |  |  |  |  |  |
| 684 | 113 | 3 | 0 | 0 | 0 | 0 | 0 | 2 | 1 |
| 691 | 113 | 0 | 0 | 0 | 0 | 0 | 0 | 0 | 0 |

Scoring: 0 = no positive IHC staining, 1 = some positive staining; 2 = moderate positive staining; 3 = extensive positive staining
